# Upper gastrointestinal cancer risk following bariatric surgery: meta-analysis

**DOI:** 10.1093/bjsopen/zrag006

**Published:** 2026-04-06

**Authors:** Heather Cooke, Rhea Harewood, Naveed Hossain, Amanda J Cross, Gwen A Murphy

**Affiliations:** Cancer Screening and Prevention Research Group, Department of Surgery and Cancer, Imperial College London, London, UK; Cancer Screening and Prevention Research Group, Department of Surgery and Cancer, Imperial College London, London, UK; Cancer Screening and Prevention Research Group, Department of Surgery and Cancer, Imperial College London, London, UK; Cancer Screening and Prevention Research Group, Department of Surgery and Cancer, Imperial College London, London, UK; Cancer Screening and Prevention Research Group, Department of Surgery and Cancer, Imperial College London, London, UK

**Keywords:** obesity, obesity-related, liver, gallbladder, oesophageal, systematic review

## Abstract

**Background:**

Obesity is a growing global epidemic, contributing to heightened risks of multiple chronic diseases, including cancer. Evidence links obesity to several malignancies, including upper gastrointestinal cancers such as oesophageal, gastric, and liver. As the need for effective weight loss intensifies, bariatric surgery is increasingly performed, and procedures have evolved, with Roux-en-Y gastric bypass and sleeve gastrectomy being the most commonly performed surgeries. Given the physiological changes after surgery, the authors conducted a systematic review and meta-analysis to examine the impact of bariatric surgery on upper gastrointestinal cancers.

**Methods:**

Eligible studies investigating bariatric surgery and upper gastrointestinal cancer incidence were identified through MEDLINE, Embase, and citation tracking up to July 2025. Studies reporting on oesophageal, gastric, liver, pancreatic, gallbladder, biliary tract, or small intestinal cancer were included. Random-effects models were used to estimate pooled relative risks (RR) and 95% confidence intervals.

**Results:**

Across 20 included studies, including 1,173,113 patients undergoing bariatric surgery and 4,600,719 patients not undergoing surgery, bariatric surgery, compared with no surgery, was inversely associated with overall upper gastrointestinal cancer risk (RR 0.58, 95% confidence interval (c.i.) 0.48 to 0.71). Specifically, inverse associations were observed for oesophageal (RR 0.63, 95% c.i. 0.40 to 0.98), liver (RR 0.47, 95% c.i. 0.35 to 0.61), and gallbladder cancer (RR 0.33, 95% c.i. 0.17 to 0.65). No significant associations were found for gastric or pancreatic cancers. There were too few studies for biliary tract and small intestinal cancers to allow meta-analyses.

**Conclusion:**

Bariatric surgery appears to reduce the risk of oesophageal, liver, and gallbladder cancers, supporting a role in both weight management and cancer prevention. Standardization in reporting surgical procedures is needed to clarify effects by bariatric surgery type.

## Teaser

Bariatric surgery is increasingly used to treat obesity and may also reduce the risk of upper gastrointestinal cancer in patients with obesity. This meta-analysis of 18 studies found inverse associations between bariatric surgery and upper gastrointestinal cancers grouped, oesophageal, liver, and gallbladder cancers. Improved reporting of surgical procedures is needed to clarify cancer risk differences by surgery type.

## Introduction

Over the past 100 years, the understanding and definition of obesity have undergone significant transformations. Initially viewed as a simple consequence of overeating and inactivity, obesity is now recognized as a complex, multifactorial condition, influenced by genetics, metabolism, environment, and socioeconomic factors. In 2017, the World Obesity Federation^1^ officially stated that obesity should be classified as a disease. In 2022, the World Health Organization^2^ estimated that one in eight people in the world were living with obesity. Additionally, in 2023–2024, 65% of adults aged 18 years and over in England were estimated to be overweight or living with obesity^3^. The combination of a complex aetiology, adverse health and economic outcomes, and a sharp rise in prevalence has led to obesity now being considered a global public health issue, resulting in significant economic and social burdens^4^.

It is important to consider the broader health implications of obesity, including its association with a higher risk of at least 13 different cancers, including upper gastrointestinal (UGI) cancers^5,6^. With obesity growing in prevalence worldwide, the effects of obesity-related cancer on public health are considerable. It remains uncertain for some cancers whether intentional weight loss can alter this risk^7^. The use of bariatric surgery has grown dramatically over the past few decades; this is in part due to improved and minimally invasive surgical techniques, and the recognition that it not only reduces weight but also results in significant improvement in comorbidities^8^. The Roux-en-Y gastric bypass (RYGB) has proven to be extremely effective in the short and long term, with excess weight loss of about 50% often achieved six months after surgery. Similar weight loss results are reported with sleeve gastrectomy (SG), which was first performed in 1993, making it one of the more recently developed techniques and technically less demanding than RYGB^9^.

Given that bariatric surgery is a leading treatment for obesity, it is important to understand the relationship between bariatric surgery and cancer risk. Numerous studies have found that bariatric surgery is associated with a significant reduction in the risk of certain cancer types, particularly hormone-related cancers including breast, endometrial, and ovarian cancers. This reduction in risk is often attributed to the substantial weight loss and metabolic improvements following surgery^10^. However, reliable evidence is lacking for UGI cancers; some research^11^ indicates that the risk of oesophageal adenocarcinoma does not decrease following bariatric surgery and may remain higher than in the general population.

The aim of this systematic review and meta-analysis is to evaluate and quantify the impact of bariatric surgery on the risk of UGI cancers, including oesophageal, gastric, liver, pancreatic, gallbladder, biliary tract, and small intestinal. Understanding the benefits of bariatric procedures, specifically with regard to UGI cancers, can help healthcare providers better advise patients on the most suitable options based on their individual risk profiles.

## Methods

This systematic review was conducted according to the PRISMA guidelines^12^ and was registered within PROSPERO (CRD420251077283). The exposure of interest was bariatric surgery, including jejunoileal bypass, gastric bypass, gastric banding, biliopancreatic diversion, duodenal switch, and sleeve gastrectomy. The outcomes include incidence of oesophageal, gastric, liver, pancreatic, gallbladder, biliary tract, and small intestinal cancers.

### Search strategy

A comprehensive literature search was conducted using the MEDLINE and Embase databases, covering the period from 1947 (inception) to 17 July 2025. Both keywords and Medical Subject Headings (MeSH) terms were employed for each database. Searches were performed with the Boolean operators AND or OR; for example, for the exposure, the following search terms were used: bariatric surger* OR metabolic surger* OR obesity surger* OR weight-loss surger* OR weight loss surger* OR gastroplast* OR sleeve gastrectom* OR gastrectom* OR gastric balloon OR gastric bypass OR Roux-en-Y gastric bypass OR Roux-en-Y loop OR gastroileal bypass OR biliopancreatic diversion OR biliopancreatic bypass OR jejunoileal bypass OR jejunocolic bypass OR intestinal bypass or duodenal switch OR gastric banding OR laparoscopic gastric banding OR pancreatobiliary bypass. The following corresponding MeSH terms were used: exp bariatric surgery/or exp intestine bypass/or exp gastroplasty/. No filters or limits were applied in the database search.

The full search strategy, including free text and MeSH terms, are included in *Table S1* (Embase) and *Table S2* (MEDLINE). A manual search for references in the included studies was conducted along with interrogation of studies used in other systematic reviews and meta-analyses on the topic of bariatric surgery and cancer risk.

### Eligibility criteria

The PICOS framework (Population, Interventions, Comparators, Outcomes, Study Design) was followed to determine study eligibility. The population was adults ≥ 18 years old who qualified for bariatric surgery. The intervention was bariatric surgery, with the comparator being patients who would qualify for bariatric surgery but did not have it. The outcome was UGI cancer incidence. The eligible study designs included randomized controlled trials, cohort studies, and case-control studies. Meta-analyses and cross-sectional studies were included in the search terms for reference scanning, but ultimately not included. Studies that explicitly included patients with a history of cancer were excluded, as were studies without a control group, case reports, commentaries, conference abstracts, animal studies, and editorials or those where cancer was diagnosed at baseline.

### Study selection

Articles identified through the literature search were imported into Covidence (Veritas Health Innovation, Melbourne, Australia). Two individual reviewers (H.C. and R.H.) performed title and abstract screening, followed by full-text screening. Any conflicts between the reviewers were resolved through discussion, and if necessary additional reviewers (A.J.C. and G.A.M.) were consulted to reach consensus.

### Data extraction

Data from each study were extracted by a single reviewer (H.C.) (*Table 1*), highlighting publication date, country, database type, follow-up time, extraction date range, and cancer outcome of interest, as well as measures of relative risk, such as hazard ratio, risk ratio (RR), or odds ratio with 95% confidence intervals (c.i.) (*Table S3*). Where risk estimates were not provided but sufficient data were available (for example, event counts and sample sizes), risk estimates were calculated in the form of crude RRs, not accounting for the time to event.

**Table 1:** Characteristics of included studies on bariatric surgery and upper gastrointestinal cancer

Reference	Publication date	Country	Database type	Follow-up time (years), mean(s.d.)**	Extraction date range	Cancer outcome of interest
Adams *et al*^13^	2023	USA	State registry and cancer registry	13.5 (1.7) (GBY); 2.8 (2.3) (SG); 12.1 (8.8) (NS)	1984–2018	Oesophageal, liver, and pancreatic
Hardvik Åkerström *et al*^14^	2023	Sweden, Finland, Denmark	National registry	8.1 (BS); 7.4 (NS)	1980–2019	Oesophageal and gastric
Aminian *et al*^7^	2022	USA	Healthcare	5.8 (3.4 to 8.8) (BS); 6.1 (3.9 to 8.9) (NS) (median (i.q.r.))	2004–2017	Oesophageal, liver, pancreatic, and biliary tract
Andalib *et al*^15^	2021	Canada	Administrative	7.6	2006–2017	Oesophageal
Bulsei *et al*^16^	2022	France	Discharge	5.2 (1.9) (BS); 6.0 (1.9) (NS)	2011–2017	Pancreatic
Chittajallu *et al*^17^	2023	USA	Real-world healthcare	10.0 (maximum)	NR	Oesophageal, gastric, liver, pancreatic, and gallbladder
Christou *et al*^18^	2008	USA	Single registry	5.0 (maximum)	1986–2002	Pancreatic
Hagstrom *et al*^19^	2021	Sweden	National registry	22.3 (median)	1987–2001	Liver
Hussan *et al*^20^	2020	USA	Discharge	0.8	2006–2013	Gastric, pancreatic, and biliary*
Khalid *et al*^21^	2022	USA	Insurance	5.0 (maximum)	2012–2020	Oesophageal, gastric, liver, pancreatic, and gallbladder
Lazzati *et al*^22^	2023	France	Discharge	6.1 (2.3) (BS); 5.6 (2.2) (NS)	2010–2019	Oesophageal, gastric, and oesophagogastric
Lazzati *et al*^23^	2022	France	Discharge	5.7 (2.2) (BS); 6.5 (2.3) (NS)	2010–2018	Oesophageal, gastric—non cardia, gastric—cardia, liver, and extrahepatic bile duct*, pancreatic, gallbladder, small intestine, and biliary tract
Mackenzie *et al*^24^	2018	England	Administrative	4.6 (median)	1997–2012	Oesophageal
Maret-Ouda *et al*^11^	2017	Sweden	National registry	3.7 (1.8 to 9.7) (BS); 3.5 (1.3 to 7.3) (NS) (median (i.q.r.))	NR	Oesophageal
Miller *et al*^25^	2024	USA	Single registry	6.7 (4.9) (BS); 6.6 (4.8) (NS)	2011–2019	Gastric and pancreatic
Rustgi *et al*^26^	2021	USA	Insurance	2.5 (2) (BS); 1.5 (1.4) (NS)	2007–2017	Oesophageal, gastric, liver, pancreatic, gallbladder
Schauer *et al*^27^	2019	USA	Health and insurance	4.0 (1.9) (BS); 3.4 (2) (NS)	2004–2014	Pancreatic
Tao *et al*^28^	2020	Denmark, Finland, Iceland, Norway, and Sweden	National registry	33.0 (maximum)	1980–2012	Oesophageal, liver, pancreatic, and gallbladder
Tsui *et al*^29^	2020	USA	Administrative	10.0 (maximum)	2006–2012	Oesophageal, gastric, liver, pancreatic, gallbladder, and extrahepatic bile duct*
Wei *et al*^30^	2021	China	Administrative	3.0	2006–2017	Liver, gallbladder, and extrahepatic bile duct*

s.d., standard deviation; GBY, gastric bypass; SG, sleeve gastrectomy; NS, no surgery; i.q.r., interquartile range; BS, bariatric surgery; NR, not reported; *Indicates an imprecise subtype cancer report.

### Risk of bias and quality assessment

The quality of studies was evaluated using the Newcastle–Ottawa Scale (NOS) by a single reviewer (H.C.)^31^. The NOS evaluates the risk of bias in three domains: the selection of study groups, the comparability of the groups, and the ascertainment of the outcome of interest. The total NOS score for a study ranged from 0 to 9, with higher scores indicating a lower risk of bias^32^. Studies were considered to have a low risk of bias if they achieved a NOS score of seven or higher, demonstrated in *Fig. S1*.

### Statistical analysis

The association between any bariatric surgery and risk of UGI cancer was examined using a random effects meta-analysis model. All references to SG, whether open, laparoscopic, or unspecified, were combined into a single category. Similarly, all forms of gastric bypass surgery, including gastric bypass, RYGB, and mini gastric bypass, were grouped together.

Between-study heterogeneity was assessed using the I² statistic, with values of < 25%, 25–50%, 51–75%, and > 75% interpreted as low, moderate, substantial, and high heterogeneity, respectively^33^. Additionally, Cochran’s Q statistic, a χ^2^ test, was employed to determine whether the observed variability in effect sizes exceeded what would be expected by chance. Publication bias was evaluated using funnel plots and Egger’s regression test. Prediction intervals, included under summary estiamtes in **Figs 2*-*6** and *Fig. S2*, were also examined to provide an estimate of the range within which the effect size of a future study might lie.

Several subgroup analyses were conducted to explore potential variations in the effects of bariatric surgery on UGI cancers: analyses by region (European, non-European); analyses by type of study database; analyses by follow-up time (< 5 years, 5–10 years, and > 10 years); and analyses by publication date (those published before 2020, or 2020 onwards) to capture potential changes in bariatric surgery practices and reporting standards that may have occurred over time. These subgroup analyses were only possible for UGI cancers overall, oesophageal, and pancreatic cancers as these were the three outcomes where at least two studies reported on each subgroup. Not enough data were available to carry out subgroup analyses on gastric, liver, gallbladder, biliary tract, or small intestinal cancer. Sensitivity analysis included leave-one-out testing and meta-regression. Leave-one-out testing involved removing one study at a time to assess its impact. Meta-regression examined the relationship between study characteristics and effect sizes. All analyses were conducted using R software (version 4.4.2).

## Results

**Fig. 1:**
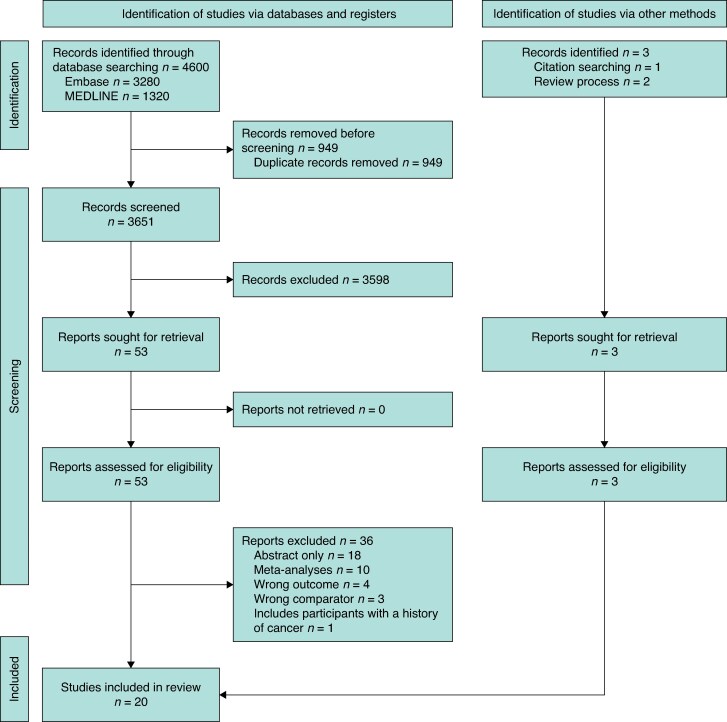
PRISMA 2020 flow diagram for new systematic reviews including searches of databases, registers, and other sources

Database searches in MEDLINE and Embase yielded 4600 results. After removing 949 duplicates, 3651 records were screened by title and abstract. Of these, 3598 were excluded, leaving 53 for full-text review. After full-text review, 17 publications remained. Additionally, one study was identified through citation tracking, and two during the review process, resulting in a total of 20 studies, comprising 1,173,113 patients undergoing bariatric surgery and 4,600,719 non-operated controls, included in the review (*Fig. 1*). Amongst these, one reported on combined oesophagogastric cancer^16^, 13 reported on oesophageal cancer^7,11,16–26^, nine reported on gastric cancer^16,18,20,21,24,26–28^ and one of these reported on gastric cardia and non-cardia separately^22^. Nine^7,13,17,19^  ^21,26,28–30^ reported on liver cancer and one^23^ reported on ‘liver and extrahepatic bile duct’ cancer. Thirteen^7,13,16–18,20,21,23,25–29^ reported on pancreatic cancer, five^17,21,23,26,28^ reported on gallbladder cancer, three^20,23,29^ reported on biliary tract cancer, two^29,30^ reported on ‘gallbladder and extrahepatic bile’ cancer, one^20^ reported on ‘gallbladder and biliary’ cancer, and one^23^ reported on small intestinal cancer. Study design included 1 case-control^19^ and 19 retrospective cohort studies^7,11,13–18,20–30^. Few studies had follow-up periods longer than 10 years, and the distribution across geographic regions was relatively balanced (*Table 1*). Detailed characteristics are given in *Table S3*.

The included studies were generally of high methodological quality (*Fig. S1*). Most NOS domains (D1–D5, D7, and D9) were consistently rated as low risk of bias. However, some concerns were noted in domains D6 and D8, primarily related to follow-up adequacy. Overall, most studies achieved low risk of bias, with only two studies^20,26^ rated as medium risk.

The overall meta-analysis, including a total of 58 extractable records relating to any of the cancers of interest from 20 studies, demonstrated a statistically significant reduction in risk of UGI cancers among those who had undergone bariatric surgery, with a summary RR of 0.58 (95% c.i. 0.48 to 071), compared with non-surgical controls (*Fig. S2*). The prediction interval ranged from 0.16 to 2.10, suggesting potential variability in future estimates. Additionally, heterogeneity was high (I² = 90%, τ² = 0.40, *P* < 0.01), reflecting high between-study variation.

Examining the association by region (*P* = 0.0002) and follow-up period (*P*  *=* 0.048) showed statistically significant difference between groups. Both non-European and European groups showed a reduction in risk of UGI cancers (RR 0.78, 95% c.i. 0.65 to 0.93; RR 0.38, 0.27 to 0.53), respectively (*Fig. S3*). Similarly, all follow-up categories— < 5 years, 5–10 years, and > 10 years—showed reductions in risk of UGI cancers (RR 0.78, 0.63 to 0.97; RR 0.48, 0.34 to 0.68; RR 0.58, 0.37 to 0.89), respectively (*Fig. S4*). No statistically significant differences were observed between the remaining subgroups investigated (*Table S4*).

**Fig. 2:**
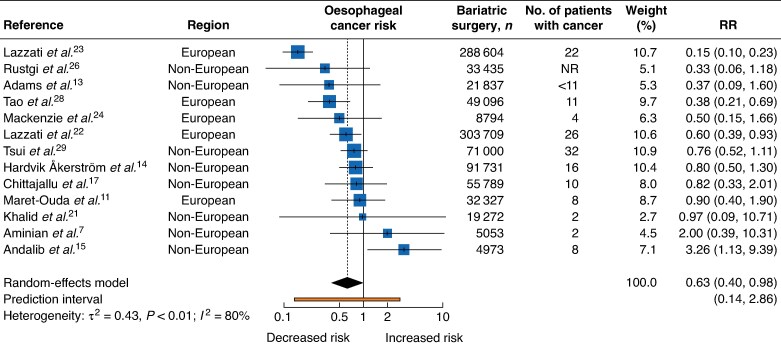
Oesophageal cancer risk following bariatric surgery RR, relative risk; NR, not reported.

A total of 13 studies evaluated the association between bariatric surgery and the risk of oesophageal cancer. Of these, three studies^22,23,28^ show a reduced risk , nine^7,11,13,14,17,21,24,26,29^ showed no statistically significant association, and one^15^ reported a significantly higher risk (*Fig. 2*). The meta-analysis for the association between bariatric surgery and oesophageal cancer showed an inverse association (RR 0.63, 95% c.i. 0.40 to 0.98); however, high heterogeneity was observed across studies (I² = 80%, τ² = 0.43, *P* < 0.01). The 95% prediction interval ranged from 0.14 to 2.86. No statistically significant differences were observed across any of the subgroups examined for oesophageal cancer (*Table S5*).

**Fig. 3:**
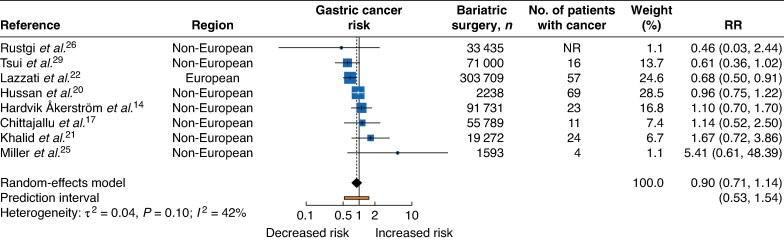
Gastric cancer risk following bariatric surgery RR, relative risk; NR, not reported.

A total of eight studies evaluated the association between bariatric surgery and gastric cancer; one study^22^ reported an inverse association, whereas the remaining seven^14,17,20,21,25,26,29^ found no statistically significant association (*Fig. 3*). Overall, the RR was 0.90 (95% c.i. 0.71 to 1.14), with moderate heterogeneity across studies (I² = 42%, τ² = 0.04, *P* = 0.101). The 95% prediction interval ranged from 0.53 to 1.54.

**Fig. 4:**
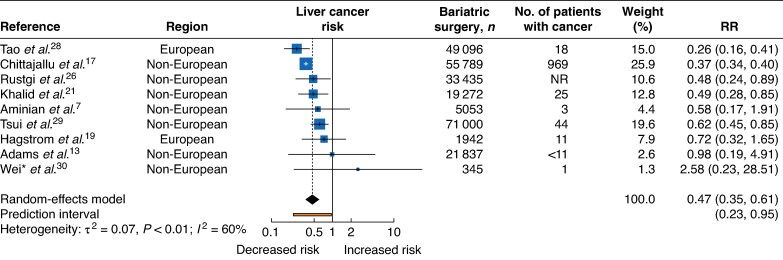
Liver cancer risk following bariatric surgery *The only study from Asia. RR, relative risk; NR, not reported.

A total of nine studies evaluated the association between bariatric surgery and liver cancer; five studies^17,21,26,28,29^ reported an inverse association, whereas the remaining four^7,13,19,30^ found no statistically significant association (*Fig. 4*). Overall, the RR was 0.47 (95% c.i. 0.35 to 0.61), with substantial heterogeneity across studies (I² = 60%, τ² = 0.07, *P* = <0.01). The 95% prediction interval ranged from 0.23 to 0.95.

**Fig. 5:**
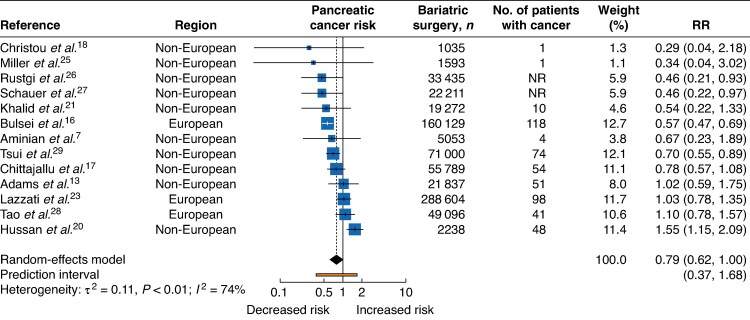
Pancreatic cancer risk following bariatric surgery RR, relative risk; NR, not reported.

A total of 13 studies evaluated the association between bariatric surgery and pancreatic cancer; 4 studies^16,26,27,29^ reported an inverse association, 8^7,13,17,18,21,23,25,28^ found no statistically significant association, and 1^20^ reported a significantly higher risk (*Fig. 5*). The overall RR was 0.79 (95% c.i. 0.62 to 1.00), with a substantial level of heterogeneity across studies (I² = 74%, τ² = 0.11, *P* = <0.01). The 95% prediction interval ranged from 0.37 to 1.68. No statistically significant differences were observed across any of the subgroups examined for pancreatic cancer (*Table S6*).

**Fig. 6:**
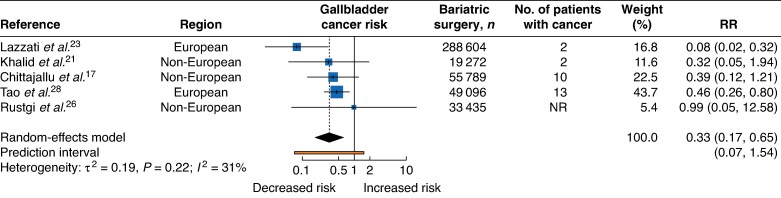
Gallbladder cancer risk following bariatric surgery RR, relative risk; NR, not reported.

A total of five studies evaluated the association between bariatric surgery and gallbladder cancer; two studies^23,28^ reported an inverse association with gallbladder cancer, whereas the remaining three^17,21,26^ found no statistically significant association (*Fig. 6*). Overall, the RR was 0.33 (95% c.i. 0.17 to 0.65) and there was moderate heterogeneity across studies (I² = 31%, τ² = 0.19, *P* = 0.22). The 95% prediction interval ranged from 0.07 to 1.54.

There were only two studies of biliary tract cancer and one of small intestinal cancer; therefore, it was not possible to conduct meta-analyses for these two outcomes. Due to the limited reporting of cancer incidence by specific surgery type, it was not feasible to assess the effects of different bariatric surgery subgroups (see *Fig. S5*).

Across all cancers, leave-one-out analyses generally supported the robustness of the findings. For pancreatic cancer, exclusion of Adams *et al*.^13^, Hussan *et al*.^20^, Lazzati *et al*.^23^, or Tao *et al*.^28^ resulted in the borderline inverse association observed, becoming statistically significant. Egger’s regression test indicated evidence of funnel plot asymmetry in the liver cancer subsite only. Funnel plots for all cancer types where estimates were meta-analysed are provided in the Supplementary material (*Figs S6–10*).

## Discussion

This analysis demonstrates a 42% lower risk of UGI cancers among individuals with obesity who had bariatric surgery compared with individuals with obesity who did not have bariatric surgery. Subgroup analysis revealed this association existed in both European and non-European populations, and in all follow-up categories. However, results should be interpreted with caution due to several studies reporting multiple cancers. When evaluating specific UGI cancer sites, an inverse association was observed between bariatric surgery and risk of oesophageal, liver, and gallbladder cancer; there was also a suggestive inverse association for pancreatic cancer. The overall finding for gastric cancer was null, and too few studies reported on small intestinal and biliary tract cancers to enable a meta-analysis to be performed. Additionally, inconsistent reporting and classification across studies precluded subgroup analyses by bariatric surgery type.

These findings align with a previous meta-analysis^34^ suggesting that bariatric surgery confers a protective effect against cancer, particularly when cancers are grouped by anatomical or metabolic classification. Previous studies have focused on either grouped cancers or a single subsite of cancer; for example, Ramai *et al*.^35^ focused specifically on hepatocellular carcinoma, whereas Wilson *et al*.^36^ investigated overall cancer risk, only reporting subsite-specific results when statistically significant. In contrast, the present review’s meta-analysis applied a focus on UGI cancers as a defined category, enabling the inclusion of both studies reporting combined cancer outcomes and those examining individual subsites that met the criteria. Some studies included in the present review also appeared in previous meta-analyses, including Lazzati *et al*.^23^ and Maret-Ouda *et al*.^11^.

Some studies, such as the systematic review and meta-analysis by Wiggins *et al*.^37^, have reported reduced overall cancer incidence among individuals who had bariatric surgery; however, when analysing specific cancer types, bariatric surgery only reduced the risk of breast cancer, underscoring the importance of looking at subsites. Chen *et al*.^38^ conducted a meta-analysis of 33 cohort studies and reported an inverse association between bariatric surgery and overall cancer, as well as specific cancer types, including breast, endometrial, and other obesity-related cancers such as liver tumours.

The present meta-analysis found bariatric surgery to be inversely associated with oesophageal, liver, and gallbladder cancer risk; these are three cancers identified as being obesity related. Based on countries in which studies were conducted, it is estimated that the majority of oesophageal cancer cases included were likely adenocarcinomas, the molecular subtype linked to obesity. Previous reports^39^ have estimated a 16% increased oesophageal adenocarcinoma risk per kg/m^2^ increase in body mass index (BMI).

The molecular mechanisms linking obesity to cancer remain unclear. Obesity may induce normal cells to become cancerous either directly through adipose tissue, inflammatory cytokines, and adipokines, or indirectly through conditions associated with a higher BMI, such as metabolic syndrome. Obesity can cause chronic inflammation, disrupting normal bodily functions^40^. Barrett’s oesophagus, the precursor lesion to oesophageal adenocarcinoma, may develop as a result of gastro-oesophageal reflux disease (GORD) arising from increased abdominal pressure with obesity^41^. Visceral fat may also contribute to oesophageal inflammation and metaplasia through reflux-independent pathways^42,43^. Obesity contributes to the progression from non-alcoholic fatty liver disease to non-alcoholic steatohepatitis and ultimately hepatocellular carcinoma^44^. There is evidence that gallbladder cancer risk is likely increased by obesity-induced gallstone formation and chronic biliary inflammation^45^. These pathophysiological links suggest that the metabolic and inflammatory improvements following bariatric surgery may reduce cancer risk across multiple sites.

Bariatric surgery can lead to significant long-term weight loss, though outcomes vary by procedure type and are influenced by anatomical and behavioural factors^46^. If weight is regained and metabolic improvements regress, the initial protective benefits of bariatric surgery may diminish. To understand better the underlying mechanisms through which bariatric surgery can reduce UGI cancers, future research should consider postoperative weight trajectories. The protective effect observed may be modified by whether patients sustain their weight loss over time. If sustained weight loss is the key driver, this would suggest a weight-dependent pathway for cancer risk reduction. Alternatively, if protection persists regardless of the levels of postoperative recurrent weight gain, other mechanisms—such as hormonal or metabolic alterations—may be involved. A major limitation to existing studies is the inability to stratify or adjust analyses by postsurgical weight change. Given that newer procedures like SG and RYGB are associated with more durable weight loss than adjustable gastric banding, stratifying by procedure type and weight change could be essential to identify which interventions offer the most effective long-term cancer protection.

Notably, only half of the included studies in this meta-analysis provided detail regarding the type of procedure performed, underscoring the need for improved reporting standards. Clapp *et al*.^47^ reported on bariatric surgery and the risk of non-hormonal cancers and found that RYGB and SG were inversely associated with overall cancer, whereas adjustable gastric banding showed no significant effect, with the authors suggesting that this may be due to the comparatively limited metabolic changes induced by banding procedures. It is increasingly clear that different types of bariatric surgery induce different anatomical and hormonal changes. Research^22^ has drawn attention to the concern that SG may lead to the onset of *de novo* GORD. Studies^48^ have reported that SG is associated with a five-fold increase in the risk of postoperative GORD compared with RYGB. This has led to some classifying bariatric surgeries into ‘reflux-prone’ (SG) and ‘reflux-protective’ (RYGB) categories. Without standardized procedural data on bariatric surgery type, comparing outcomes and developing evidence-based recommendations remain limited.

A related barrier to comparative research is the inconsistent terminology in reporting of bariatric procedures. Abbreviations for surgery type are often used without standard definitions. For example, in some instances ‘GBY’ would refer to one-anastomosis gastric bypass and in others RYGB gastric bypass. Alternatively, it could be used to group any type of gastric bypass. This lack of uniformity hinders subgroup analysis. Establishing standardized reporting—akin to ICD-10 coding and incorporating existing surgical coding systems such as the Operating Procedure Codes Supplement—could greatly enhance data quality.

It remains to be seen how glucagon-like peptide-1 (GLP-1) receptor agonists may alter the bariatric surgery landscape and the consequence of these changes with regard to subsequent cancer incidence. A recent retrospective cohort study^49^ found that a reduction in cancer risk among patients using GLP-1 receptor agonists was comparable to that observed in bariatric surgery patients, despite weight loss being inferior. This suggests that mechanisms beyond weight loss may contribute to the observed protective effect. Whereas further research is needed from well powered studies, the widespread use of GLP-1 receptor agonists among the general population may make studies difficult to undertake.

A strength of this meta-analysis was the inclusion of both broad and site-specific cancer outcomes. This approach provided a clearer understanding of how bariatric surgery may affect individual UGI cancers rather than treating all cancers as a single outcome. The small number of specific cancer cases in some studies contributed to wide confidence intervals. Nonetheless, subsite specific analysis is important due to differing epidemiology, risk factors, and biological behaviour, and different organ responses to bariatric surgery. Although examined by organ subsite, the authors were unable to distinguish between subtypes within an organ, such as oesophageal adenocarcinoma and squamous cell carcinoma, or gastric cardia and non-cardia cancers. In addition, the authors were unable to examine differences between types of bariatric surgery due to insufficient detail in the reported data. The geographic scope was also limited, with only one study conducted in an Asian population.

There may be potential confounding factors that the present analysis is unable to address. In non-randomized studies, a major concern is selection bias: patients who undergo surgery may differ systematically from those who do not, in terms of comorbidities, age, health beliefs, and treatment adherence. These differences can influence both the likelihood of receiving surgery and subsequent outcomes. Whereas some studies attempt to mitigate this through matched cohort designs and adjusted analyses, the extent and type of adjustment vary; some report only crude case counts, leaving room for residual confounding. Earlier studies may not have been aware of, or accounted for, confounders as rigorously as more recent ones, contributing to potential bias. Further confounding may arise from differences in preoperative preparation and postoperative care. Patients who undergo bariatric surgery typically receive multidisciplinary support and undergo various assessments, including that for diabetes management, which may contribute to health improvements before surgery. In contrast, individuals who do not undergo surgery may not receive comparable medical support to initiate lifestyle changes. Additionally, cancer rates among patients who undergo bariatric surgery may appear higher in some cases due to increased routine imaging and follow-up care^50^, which may inflate incidence rates and introduce detection bias. Furthermore, many included studies had relatively short follow-up periods, which may be insufficient to detect long-term cancer outcomes, highlighting the need for extended follow-up in future research^51^. Finally, there were not enough data available to assess differential risks by age, sex, or other demographic characteristics.

This meta-analysis reported that bariatric surgery is inversely associated with oesophageal, liver, and gallbladder cancer. However, relationships between bariatric surgery and cancer risk are complex and likely influenced by multiple factors, including the type of bariatric procedure, the sustainability of postoperative weight loss, baseline BMI, and patient-specific behavioural, psychological, anatomical, and neurobiological characteristics. It is not clear whether significant recurrent weight gain in some individuals may diminish the long-term cancer protection initially conferred by surgery.

The findings of this review highlight the need for standardized data capture, especially accurate documentation of the type of bariatric surgery performed. Only with high-quality, granular data can researchers and clinicians determine which surgical approaches offer the greatest long-term benefits with the fewest complications. Incorporating bariatric surgery into broader cancer prevention strategies, alongside mental health care, nutritional support, and personalized follow-up, offers a promising, multifaceted path forward for individuals with obesity.

## Supplementary Material

zrag006_Supplementary_Data

## Data Availability

Extracted data available upon request.
