# Supplementary material for: Upper gastrointestinal cancer risk following bariatric surgery: meta-analysis
Source: BJS Open. 2026 Apr 6;10(2):zrag006. doi: 10.1093/bjsopen/zrag006 (PMC13056541; doi:10.1093/bjsopen/zrag006)
Supplement: zrag006_Supplementary_Data [file zrag006_supplementary_data.docx]

**Upper gastrointestinal cancer risk following bariatric surgery: meta-analysis**

Heather Cooke^1^, Rhea Harewood^1^, Naveed Hossain^1^, Amanda J Cross^1^, Gwen A Murphy^1^

^1^Cancer Screening and Prevention Research Group, Department of Surgery and Cancer, Imperial College London, London, UK

**Corresponding author:**

Heather Cooke

Department of Surgery and Cancer, Imperial College London

[h.cooke@imperial.ac.uk](mailto:h.cooke@imperial.ac.uk)

**ORCID ID: 0009-0008-5535-2042**

**Supplementary Materials - Index**

|  |  |
| --- | --- |
| **Supplementary Figures and Tables** |  |
| Table S1. Search Strategy Embase  Table S2. Search strategy MEDLINE  Table S3. Study characteristics  Table S4. Subgroup analysis of upper gastrointestinal cancer risk following bariatric surgery: Risk ratio (RR) and 95% confidence intervals (CI) by database type, follow-up period, and publication date  Table S5. Subgroup analysis of oesophageal cancer risk following bariatric surgery: Risk ratio (RR) and 95% confidence intervals (CI) by follow-up period, publication date, and region  Table S6. Subgroup analysis of pancreatic cancer risk following bariatric surgery: Risk ratio (RR) and 95% confidence intervals (CI) by follow-up period, publication date, and region    Figure S1. Risk of bias assessment (Newcastle-Ottawa Scale)  Figure S2. Bariatric surgery and overall upper gastrointestinal risk by cancer subtype  Figure S3. Upper gastrointestinal cancer risk following bariatric surgery: subgroup analysis by region  Figure S4. Upper gastrointestinal cancer risk following bariatric surgery: subgroup analysis by follow-up category  Figure S5. Distribution of studies reporting upper gastrointestinal cancer incidence after bariatric surgery by cancer type and surgery type  Figure S6. Funnel plot of publication bias for oesophageal cancer studies  Figure S7. Funnel plot of publication bias for gastric cancer studies  Figure S8. Funnel plot of publication bias for liver cancer studies  Figure S9. Funnel plot of publication bias for pancreatic cancer studies  Figure S10. Funnel plot of publication bias for gallbladder cancer studies | *Page 3*  *Page 4*  *Page 5*  *Page 7*  *Page 7*  *Page 7*  *Page 8*  *Page 9*  *Page 10*  *Page 11*  *Page 12*  *Page 13*  *Page 14*  *Page 15*  *Page 16*  *Page 17* |

**Supplementary Figures and Tables**

**Table S1** Search strategy for Embase

|  | # | Search | Concepts |
| --- | --- | --- | --- |
| Free Text | 1 | (upper gastrointestinal or upper GI or UGI or oesophag* or esophag* or gastric or stomach or cardia or non cardia or non-cardia or excluded stomach or pancrea* or small intestine or duodenum or jejunum or ileum or gall bladder or liver or hepatocellular or biliary duct) adj4 (cancer or tumo?r or carcinoma* or adenocarcinoma* or neoplas* or metastas* or lymphoma* or malignanc* or squamo?s cell or oncogenesis or dysplasia) | Upper Gastrointestinal Cancer |
| MeSH | 2 | exp pancreatobiliary cancer/ OR exp esophagus tumor/ OR exp small intestine tumor/ OR exp stomach tumor/ OR exp liver tumor/ |  |
|  | 3 | 1 OR 2 |  |
| Free Text | 4 | bariatric surger* or metabolic surger* or obesity surger* or weight-loss surger* or weight loss surger* or gastroplast* or sleeve gastrectom* or gastrectom* or gastric balloon or gastric bypass or Roux-en-Y gastric bypass or Roux-en-Y loop or gastroileal bypass or biliopancreatic diversion or biliopancreatic bypass or jejunoileal bypass or jejunocolic bypass or intestinal bypass or duodenal switch or gastric banding or laparoscopic gastric banding or pancreatobiliary bypass | Bariatric Surgery |
| MeSH | 5 | exp bariatric surgery/ or exp intestine bypass/ or exp gastroplasty/ |  |
|  | 6 | 4 OR 5 |  |
|  | 7 | 3 AND 6 | Upper GI Cancer AND Bariatric Surgery |
| Free Text | 8 | obes* or weight loss or weight-loss or overweight or over-weight or bmi or body mass index | Obesity |
| MeSH | 9 | exp obesity/ or body mass/ |  |
|  | 10 | 8 OR 9 |  |
|  | 11 | 3 AND 6 AND 10 | Upper GI Cancer AND Bariatric Surgery AND Obesity |
| Free Text | 12 | (cohort adj4 study) or case control or cross-sectional or case series or retrospective stud* or prospective stud* or longitudinal stud* or observational stud* or comparative stud* or follow-up stud* | Upper GI Cancer AND Bariatric Surgery AND Obesity AND Study Type |
| MeSh | 13 | follow up/ or longitudinal study/ or prospective study/ or retrospective study/ or clinical trial/ or observational study/ or case control study/ or cross-sectional study/ or meta analysis/ or cohort analysis/ |  |
|  | 14 | 12 OR 13 |  |
|  | 15 | 3 AND 6 AND 10 AND 14 |  |

**Table S2** Search strategy for MEDLINE

|  | # | Search | Concepts |
| --- | --- | --- | --- |
| Free Text | 1 | (upper gastrointestinal or upper GI or UGI or oesophag* or esophag* or gastric or stomach or cardia or non cardia or non-cardia or excluded stomach or pancrea* or small intestine or duodenum or jejunum or ileum or gall bladder or liver or hepatocellular or biliary duct) adj4 (cancer or tumo?r or carcinoma* or adenocarcinoma* or neoplas* or metastas* or lymphoma* or malignanc* or squamo?s cell or oncogenesis or dysplasia) | Upper Gastrointestinal Cancer |
| MeSH | 2 | Biliary Tract Neoplasms/ or Esophageal Neoplasms/ or Duodenal Neoplasms/ or Ileal Neoplasms/ or Jejunal Neoplasms/ or Stomach Neoplasms/ or Liver Neoplasms/ or Pancreatic Neoplasms/ |  |
|  | 3 | 1 OR 2 |  |
| Free Text | 4 | bariatric surger* or metabolic surger* or obesity surger* or weight-loss surger* or weight loss surger* or gastroplast* or sleeve gastrectom* or gastrectom* or gastric balloon or gastric bypass or Roux-en-Y gastric bypass or Roux-en-Y loop or gastroileal bypass or biliopancreatic diversion or biliopancreatic bypass or jejunoileal bypass or jejunocolic bypass or intestinal bypass or duodenal switch or gastric banding or laparoscopic gastric banding or pancreatobiliary bypass | Bariatric Surgery |
| MeSH | 5 | Bariatric Surgery/ or Gastric Bypass/ or Gastroplasty/ or Jejunoileal Bypass/ or Lipectomy/ or Anastomosis, Roux-en-Y/ or Biliopancreatic Diversion/ or Gastrostomy/ |  |
|  | 6 | 4 OR 5 |  |
|  | 7 | 3 AND 6 | Upper GI Cancer AND Bariatric Surgery |
| Free Text | 8 | obes* or weight loss or weight-loss or overweight or over-weight or bmi or body mass index | Obesity |
| MeSH | 9 | Overweight/ OR Obesity/ OR Obesity, Morbid/ OR Body Mass Index/ |  |
|  | 10 | 8 OR 9 |  |
|  | 11 | 3 AND 6 AND 10 | Upper GI Cancer AND Bariatric Surgery AND Obesity |
| Free Text | 12 | (cohort adj4 study) or case control or cross-sectional or case series or retrospective stud* or prospective stud* or longitudinal stud* or observational stud* or comparative stud* or follow-up stud* | Study type |
| MeSH | 13 | Follow-Up Studies/ or Longitudinal Studies/ or Prospective Studies/ or Retrospective Studies/ or Clinical Trial/ or Observational Study/ or Case-Control Studies/ or Cross-Sectional Studies/ or Meta-Analysis/ or Cohort Studies/ |  |
|  | 14 | 12 OR 13 |  |
|  | **15** | **3 AND 6 AND 10 AND 14** | **Upper GI Cancer, Bariatric Surgery, Obesity and Study Type** |

**Table S3** Characteristics of studies included in the meta-analysis

| Author (reference number) | Study Design | Bariatric Surgery, n | No Surgery, n | Cancer Type | Number of cancers in Bariatric Surgery Group | Number of cancers in No Surgery Group | Risk |
| --- | --- | --- | --- | --- | --- | --- | --- |
| Adams et al (17) | Retrospective cohort | 21837 | 21837 | Oesophageal | <11 | 11 | HR |
| Adams et al (17) | Retrospective cohort | 21837 | 21837 | Liver | <11 | 13 | HR |
| Adams et al (17) | Retrospective cohort | 21837 | 21837 | Pancreatic | 51 | 42 | HR |
| Akerstrom et al (18) | Retrospective cohort | 91731 | 693799 | Oesophageal | 16 | 274 | HR |
| Akerstrom et al (18) | Retrospective cohort | 91731 | 693799 | Gastric | 23 | 296 | HR |
| Aminian et al (7) | Retrospective cohort | 5053 | 25265 | Oesophageal | 2 | 5 | RR* |
| Aminian et al (7) | Retrospective cohort | 5053 | 25265 | Liver | 3 | 26 | RR* |
| Aminian et al (7) | Retrospective cohort | 5053 | 25265 | Pancreatic | 4 | 30 | RR* |
| Aminian et al (7) | Retrospective cohort | 5053 | 25265 | Biliary Tract | 2 | 9 | RR* |
| Andalib (19) | Retrospective cohort | 4973 | 12159 | Oesophageal | 8 | 6 | RR* |
| Bulsei et al (32) | Retrospective cohort | 160129 | 1262804 | Pancreatic | 118 | 4478 | HR |
| Chittajallu et al (20) | Retrospective cohort | 55789 | 55789 | Oesophageal | 10 | 10 | HR |
| Chittajallu et al (20) | Retrospective cohort | 55789 | 55789 | Gastric | 11 | 12 | HR |
| Chittajallu et al (20) | Retrospective cohort | 55789 | 55789 | Liver | 969 | 2198 | HR |
| Chittajallu et al (20) | Retrospective cohort | 55789 | 55789 | Pancreatic | 54 | 86 | HR |
| Chittajallu et al (20) | Retrospective cohort | 55789 | 55789 | Gallbladder | 10 | 10 | HR |
| Christou et al (33) | Retrospective cohort | 1035 | 5746 | Pancreatic | 1 | 19 | RR* |
| Hagstrom et al (30) | Case-control | 1942 | 1980 | Liver | 11 | 15 | HR |
| Hussan (28) | Retrospective cohort | 238 | 28041 | Gastric | 69 | 900 | RR* |
| Hussan (28) | Retrospective cohort | 238 | 28041 | Pancreatic | 48 | 387 | RR* |
| Hussan (28) | Retrospective cohort | 238 | 28041 | Gallbladder and biliary | 61 | 487 | RR* |
| Khalid et al (21) | Retrospective cohort | 19272 | 9363 | Oesophageal | 2 | 1 | RR* |
| Khalid et al (21) | Retrospective cohort | 19272 | 9363 | Gastric | 24 | 7 | RR* |
| Khalid et al (21) | Retrospective cohort | 19272 | 9363 | Liver | 25 | 25 | RR* |
| Khalid et al (21) | Retrospective cohort | 19272 | 9363 | Pancreatic | 10 | 9 | RR* |
| Khalid et al (21) | Retrospective cohort | 19272 | 9363 | Gallbladder | 2 | 3 | RR* |
| Lazzati et al (16) | Retrospective cohort | 303709 | 605140 | Oesophageal | 26 | 86 | RR* |
| Lazzati et al (16) | Retrospective cohort | 303709 | 605140 | Gastric | 57 | 168 | RR* |
| Lazzati et al (16) | Retrospective cohort | 303709 | 605140 | Oesophogastric | 83 | 254 | HR |
| Lazzati et al (22) | Retrospective cohort | 288604 | 851743 | Oesophageal | 22 | 435 | RR* |
| Lazzati et al (22) | Retrospective cohort | 288604 | 851743 | Gastric - non cardia | 31 | 544 | RR* |
| Lazzati et al (22) | Retrospective cohort | 288604 | 851743 | Gastric - cardia | 15 | 203 | RR* |
| Lazzati et al (22) | Retrospective cohort | 288604 | 851743 | Liver and extrahepatic bile duct | 40 | 914 | RR* |
| Lazzati et al (22) | Retrospective cohort | 288604 | 851743 | Pancreatic | 98 | 1328 | HR |
| Lazzati et al (22) | Retrospective cohort | 288604 | 851743 | Gallbladder | 2 | 75 | RR* |
| Lazzati et al (22) | Retrospective cohort | 288604 | 851743 | Small intestine | 19 | 201 | RR* |
| Lazzati et al (22) | Retrospective cohort | 288604 | 851743 | Biliary Tract | 10 | 184 | RR* |
| Mackenzie et al (23) | Retrospective cohort | 8794 | 8794 | Oesophageal | 4 | 8 | OR |
| Maret-Ouda et al (11) | Retrospective cohort | 32327 | 123695 | Oesophageal | 8 | 53 | HR |
| Miller et al (29) | Retrospective cohort | 1593 | 2156 | Gastric | 4 | 1 | RR |
| Miller et al (29) | Retrospective cohort | 1593 | 2156 | Pancreatic | 1 | 4 | RR |
| Rustgi et al (24) | Retrospective cohort | 33435 | 64655 | Oesophageal | NR | NR | HR |
| Rustgi et al (24) | Retrospective cohort | 33435 | 64655 | Gastric | NR | NR | HR |
| Rustgi et al (24) | Retrospective cohort | 33435 | 64655 | Liver | NR | NR | HR |
| Rustgi et al (24) | Retrospective cohort | 33435 | 64655 | Pancreatic | NR | NR | HR |
| Rustgi et al (24) | Retrospective cohort | 33435 | 64655 | Gallbladder | NR | NR | HR |
| Schauer et al (34) | Retrospective cohort | 22211 | 66481 | Pancreatic | NR | NR | HR |
| Tao et al (25) | Retrospective cohort | 49096 | 436476 | Oesophageal | 11 | 260 | RR* |
| Tao et al (25) | Retrospective cohort | 49096 | 436476 | Liver | 18 | 617 | RR* |
| Tao et al (25) | Retrospective cohort | 49096 | 436476 | Pancreatic | 41 | 808 | HR |
| Tao et al (25) | Retrospective cohort | 49096 | 436476 | Gallbladder | 13 | 251 | RR* |
| Tsui et al (26) | Retrospective cohort | 71000 | 323197 | Oesophageal | 32 | 191 | RR* |
| Tsui et al (26) | Retrospective cohort | 71000 | 323197 | Gastric | 16 | 120 | RR* |
| Tsui et al (26) | Retrospective cohort | 71000 | 323197 | Liver | 44 | 324 | RR* |
| Tsui et al (26) | Retrospective cohort | 71000 | 323197 | Pancreatic | 74 | 481 | RR* |
| Tsui et al (26) | Retrospective cohort | 71000 | 323197 | Gallbladder and extrahepatic bile duct | 34 | 138 | RR* |
| Wei et al (31) | Retrospective cohort | 345 | 1599 | Liver | 1 | 2 | HR |
| Wei et al (31) | Retrospective cohort | 345 | 1599 | Gallbladder and extrahepatic bile duct | 1 | 1 | HR |

Abbreviations: BS, bariatric surgery; HR, hazard ratio; NR, not reported; NS, no-surgery; OR, odds ratio; RR; risk ratio *Crude risk ratio calculated

**Table S4** Subgroup analysis of upper gastrointestinal cancer risk following bariatric surgery: Risk ratio (RR) and 95% confidence intervals by database type and publication date

| Category | Subgroup | RR | 95%-CI | Subgroup difference (p value) |
| --- | --- | --- | --- | --- |
| Database Type | State registry and cancer registry | 0.91 | [0.56; 1.47] | **0.285** |
|  | National registry | 0.64 | [0.43; 0.95] |  |
|  | Healthcare | 0.60 | [0.29; 1.23] |  |
|  | Administrative | 0.81 | [0.63; 1.03] |  |
|  | Discharge | 0.42 | [0.26; 0.98] |  |
|  | Real-world data platform | 0.62 | [0.39; 0.98] |  |
|  | Single-registry | 0.79 | [0.13; 4.99] |  |
|  | Insurance | 0.57 | [0.40; 0.80] |  |
|  | Healthcare and insurance | 0.46 | [0.22; 0.97] |  |
| Publication date | After 2020 | 0.53 | [0.41; 0.68] | **0.091** |
|  | 2020 or before | 0.73 | [0.56; 0.95] |  |

**Table S5** Subgroup analysis of oesophageal cancer risk following bariatric surgery: Risk ratio (RR) and 95% confidence intervals (CI) by follow-up period, publication date and region

| Category | Subgroup | RR | 95%-CI | Subgroup difference (p value) |
| --- | --- | --- | --- | --- |
| Follow-up (years) | <5 | 0.74 | [0.54; 1.01] | **0.107** |
|  | 5-10 | 0.76 | [0.32; 1.81] |  |
|  | >10 | 0.38 | [0.22; 0.65] |  |
| Publication date | After 2020 | 0.66 | [0.34; 1.27] | **0.889** |
|  | 2020 or before | 0.62 | [0.41; 0.95] |  |
| Region | Non-European | 0.83 | [0.64; 1.07] | **0.051** |
|  | European | 0.41 | [0.22; 0.79] |  |

**Table S6** Subgroup analysis of pancreatic cancer risk following bariatric surgery: Risk ratio (RR) and 95% confidence intervals (CI) by follow-up period, publication date and region

| Category | Subgroup | RR | 95%-CI | Subgroup difference (p value) |
| --- | --- | --- | --- | --- |
| Follow-up (years) | <5 | 0.68 | [0.42; 1.11] | **0.139** |
|  | 5-10 | 0.74 | [0.54; 1.01] |  |
|  | >10 | 1.08 | [0.80; 1.44] |  |
| Publication date | After 2020 | 0.73 | [0.57; 0.94] | **0.530** |
|  | 2020 or before | 0.86 | [0.54; 1.39] |  |
| Region | Non-European | 0.75 | [0.55; 1.02] | **0.639** |
|  | European | 0.85 | [0.55; 1.30] |  |

**Figure S1** Risk of bias assessment (Newcastle-Ottawa Scale)


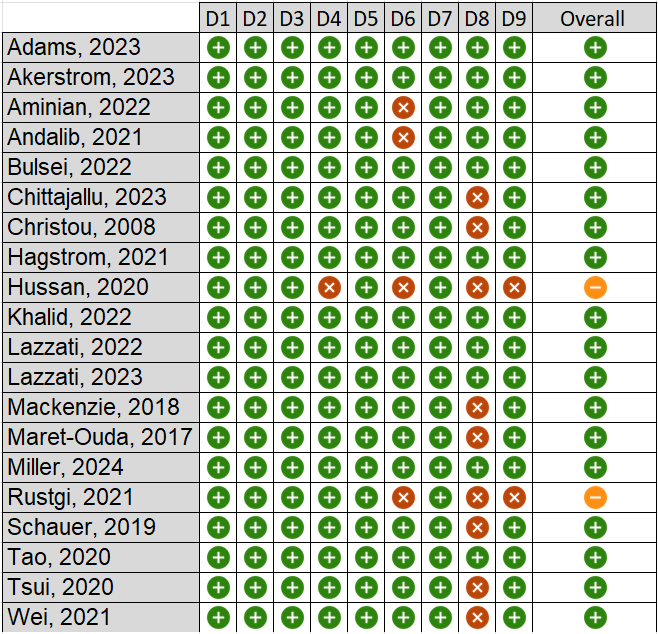


Abbreviations: D, Domain

Domain definitions: D1, selection of study groups; D2, comparability of groups; D3, exposure for case-controlstudies/outome for cohort studies; D4, demonstration that outcome of interest was not present at start/selection of controls; D5, comparability of cohort cases/controls based on design or analysis (age and sex); D6, additional comparability factors (e.g., BMI, socioeconomic status, alcohol consumption); D7, assessment of outcome/exposure; D8, adequacy of follow-up for outcome to occur (>5 yeasrs)/non-response rate; D9, adequacy of follow-up of cohorts/ascertainment of exposure for case-control

Each domain is scored following a colour scheme: Green, meets criteria; Red, misses criteria

The overall score is represented a traffic light colour scheme: Green, low risk of bias (score 7 to 9); Orange, moderate risk of bias (score 4-6); Red, high risk of bias (score <4)

**Figure S2** Bariatric surgery and overall upper gastrointestinal risk by cancer subtype

**
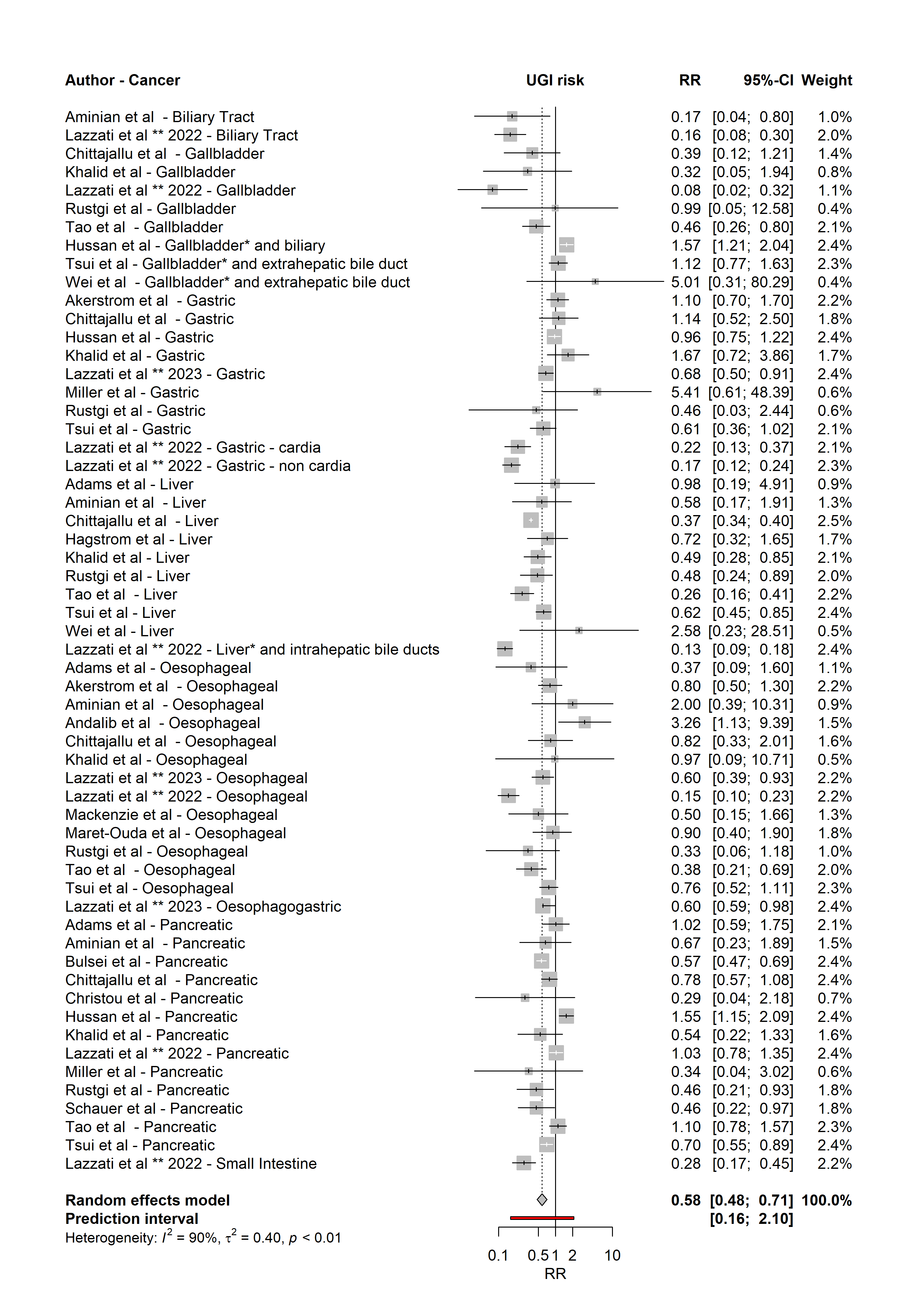
**

Abbreviations: CI, confidence interval; RR, risk ratio; UGI, upper gastrointestinal cancer.

* Indicates an imprecise subtype cancer report; **Year, highlights which Lazzati paper is being presented (2022 or 2023)

**Figure S3** Upper gastrointestinal cancer risk following bariatric surgery: subgroup analysis by region

**
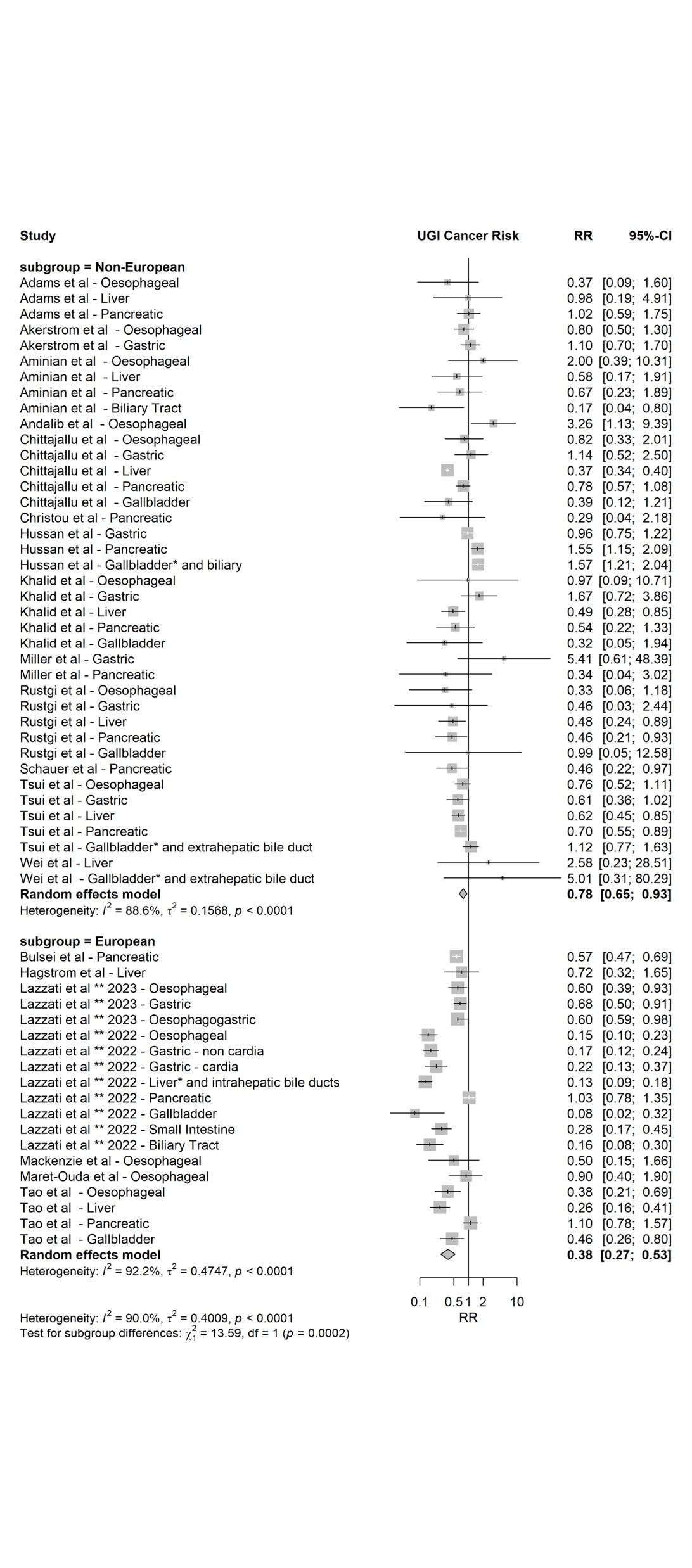
**

Abbreviations: CI, confidence interval; RR, risk ratio; UGI, upper gastrointestinal cancer

* Indicates an imprecise subtype cancer report; **Year, highlights which Lazzati paper is being presented (2022 or 2023)

**Figure S4** Upper gastrointestinal cancer risk following bariatric surgery: subgroup analysis by duration of follow-up category


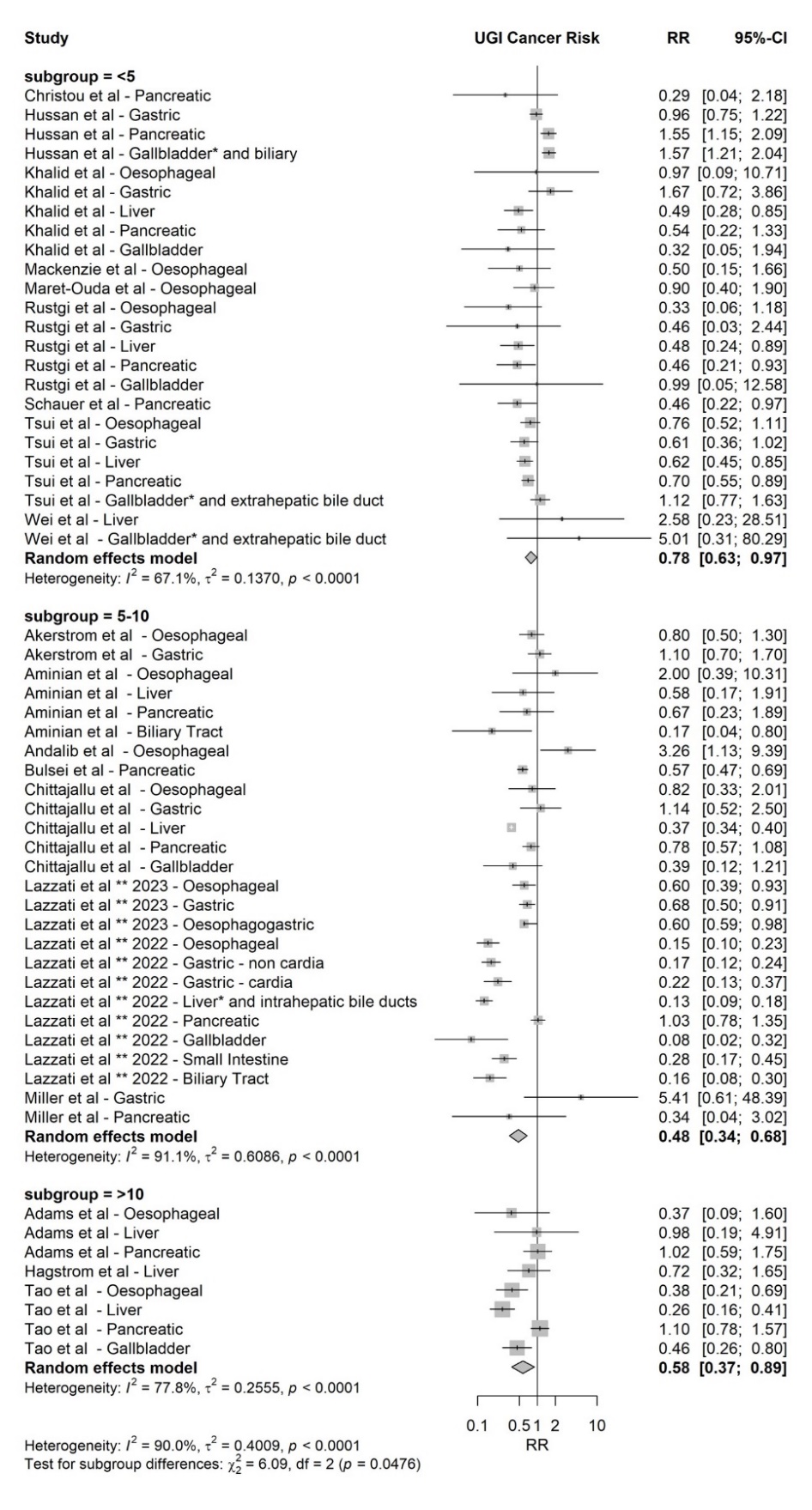


Abbreviations: CI, confidence interval; RR, risk ratio; UGI, upper gastrointestinal cancer

* Indicates an imprecise subtype cancer report; **Year, highlights which Lazzati paper is being presented (2022 or 2023)

**Figure S5** Distribution of studies reporting upper gastrointestinal cancer incidence after bariatric surgery by cancer type and surgery type

**
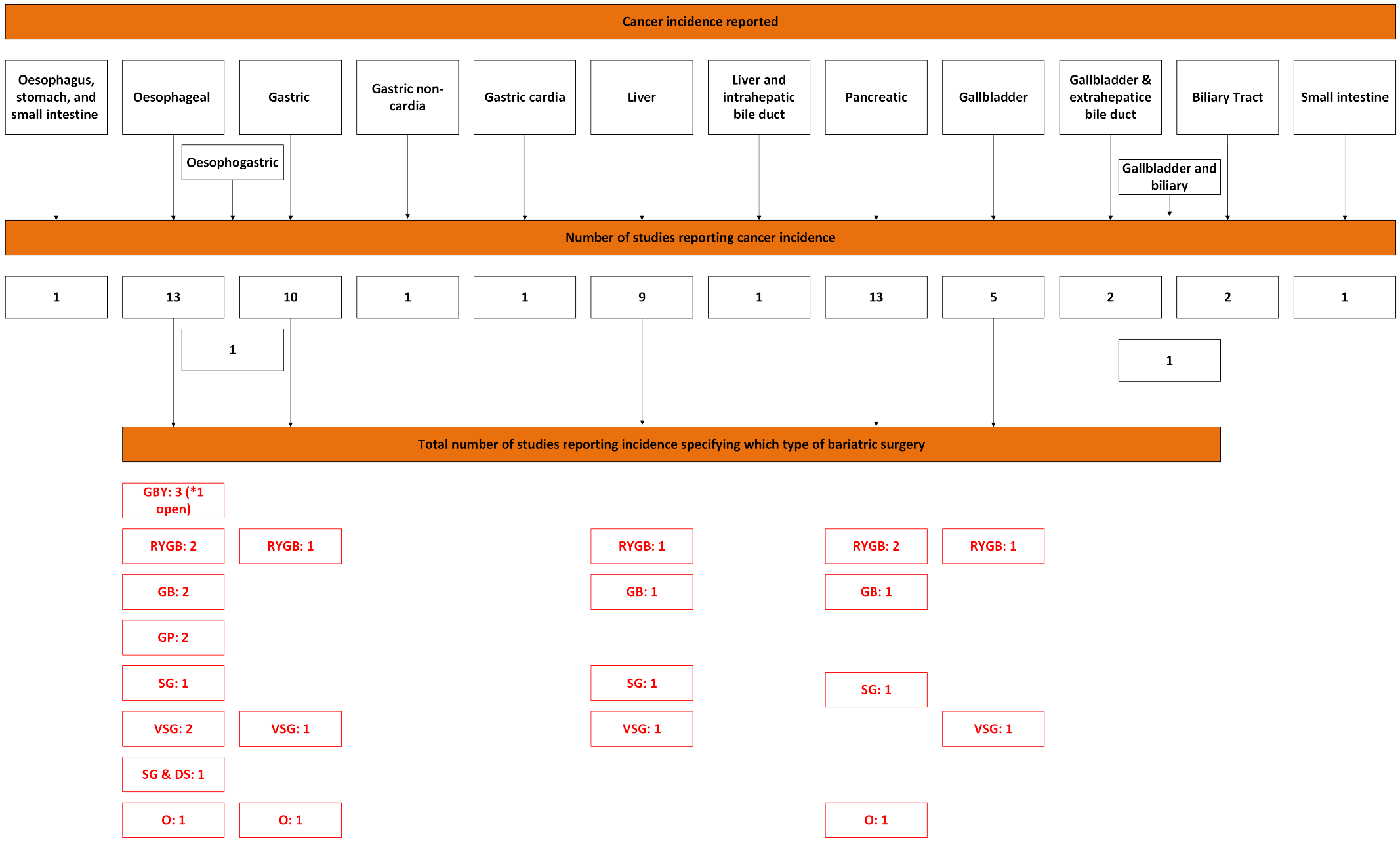
**

Abbreviations: GB, gastric banding; GBY, gastric bypass; GP, gastroplasty; O, other; RYGB, Roux-en-Y gastric bypass; SG, sleeve gastrectomy; SG & DS, sleeve gastrectomy and duodenal switch; VSG, vertical sleeve gastrectomy**.**

**Figure S6** Funnel plot of publication bias for oesophageal cancer incidence following bariatric surgery studies

**
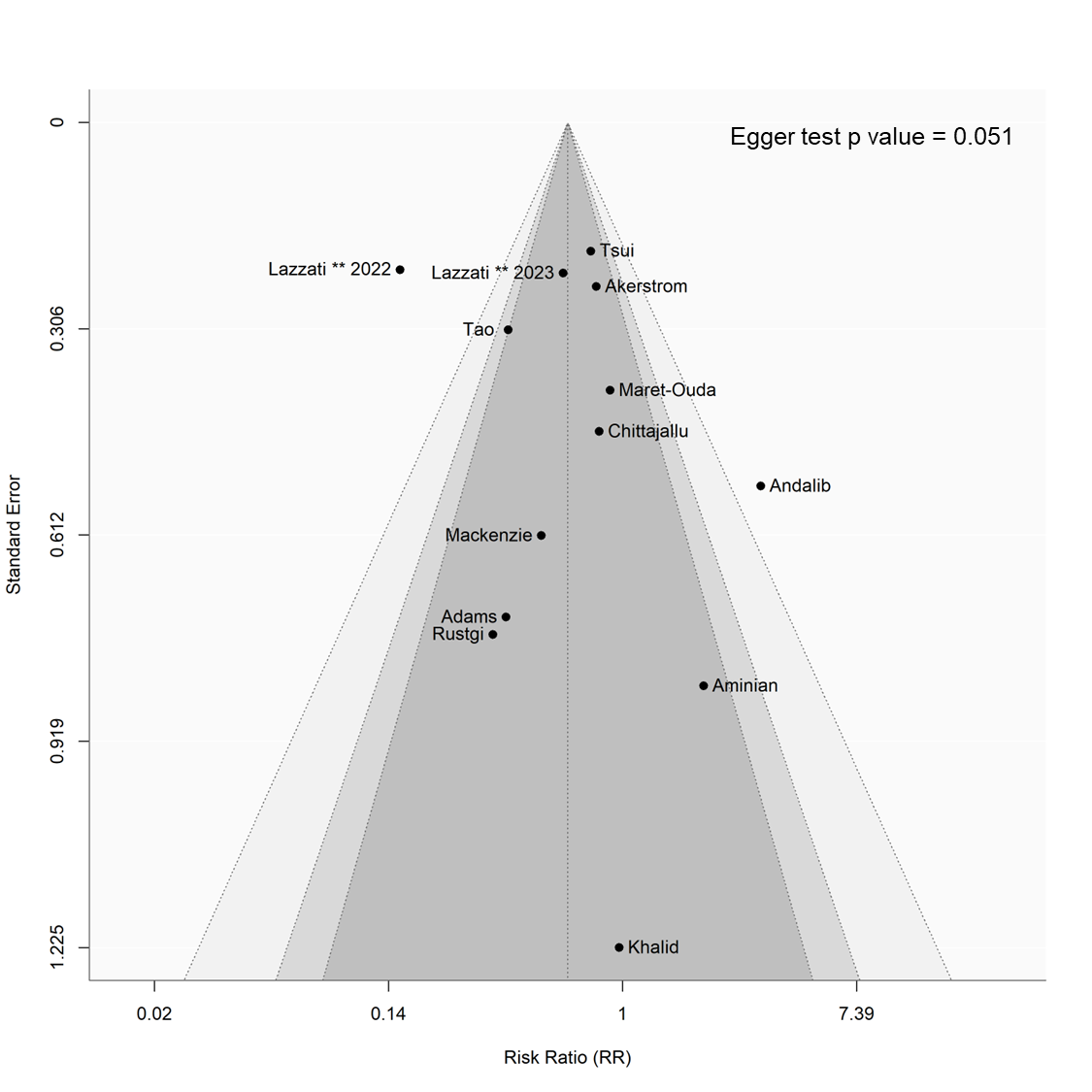
**

**Year, highlights which Lazzati paper is being presented (2022 or 2023)

**Figure S7** Funnel plot of publication bias for gastric cancer incidence following bariatric surgery studies


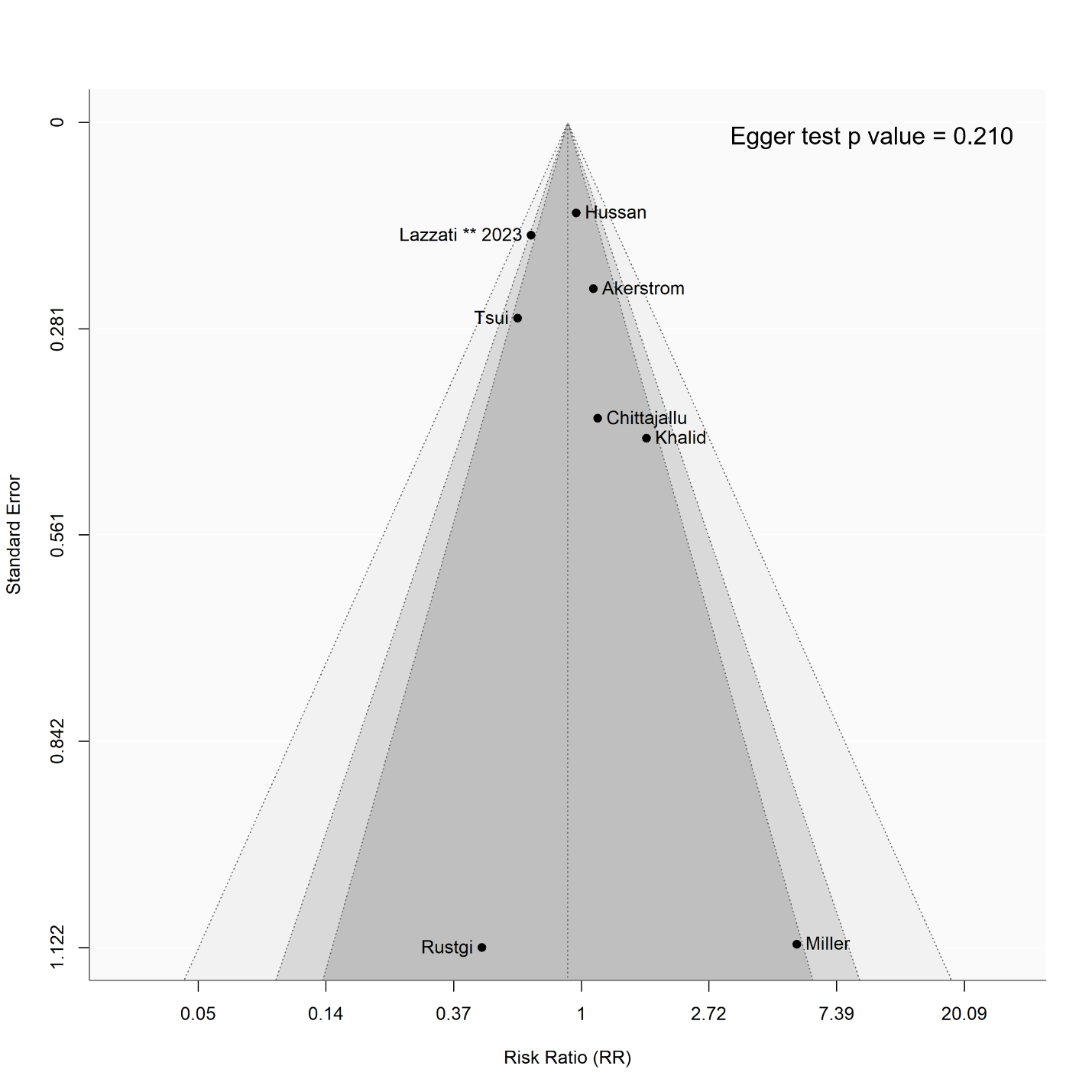


**Year, highlights which Lazzati paper is being presented (2022 or 2023)

**Figure S8** Funnel plot of publication bias for liver cancer incidence following bariatric surgery studies

**
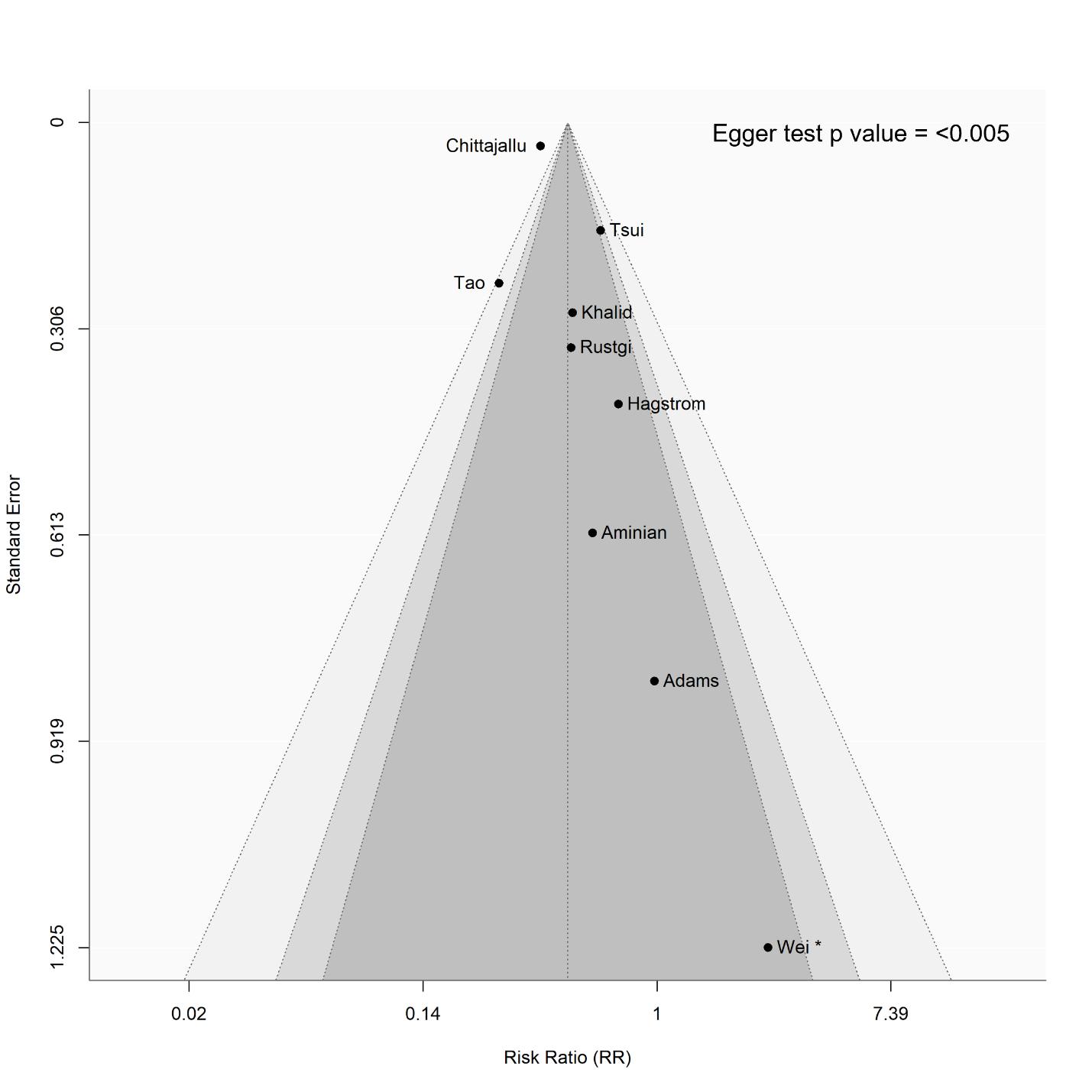
**

**Figure S9** Funnel plot of publication bias for pancreatic cancer incidence following bariatric surgery studies

**
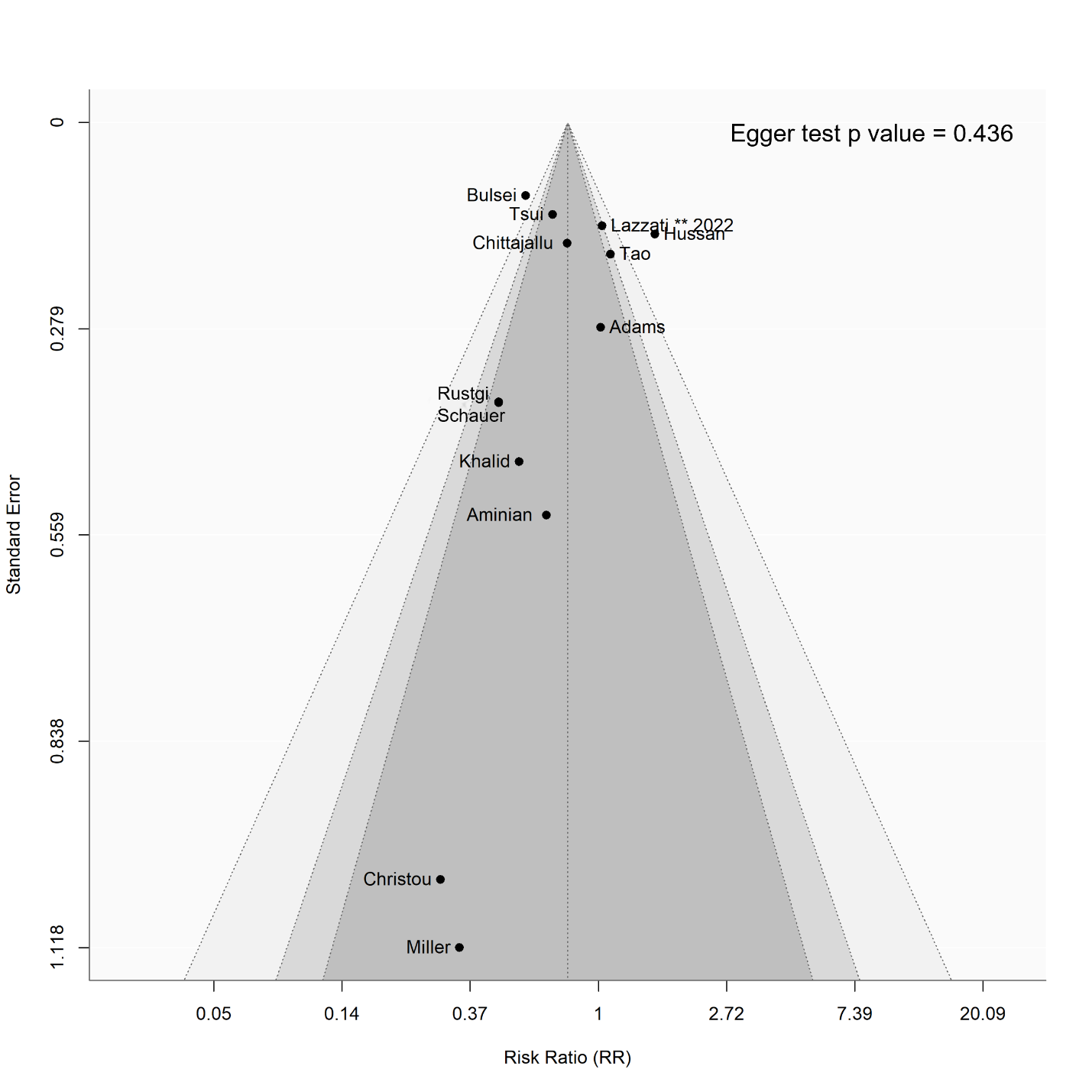
**

**Year, highlights which Lazzati paper is being presented (2022 or 2023)

**Figure S10** Funnel plot of publication bias for gallbladder cancer incidence following bariatric surgery studies

**
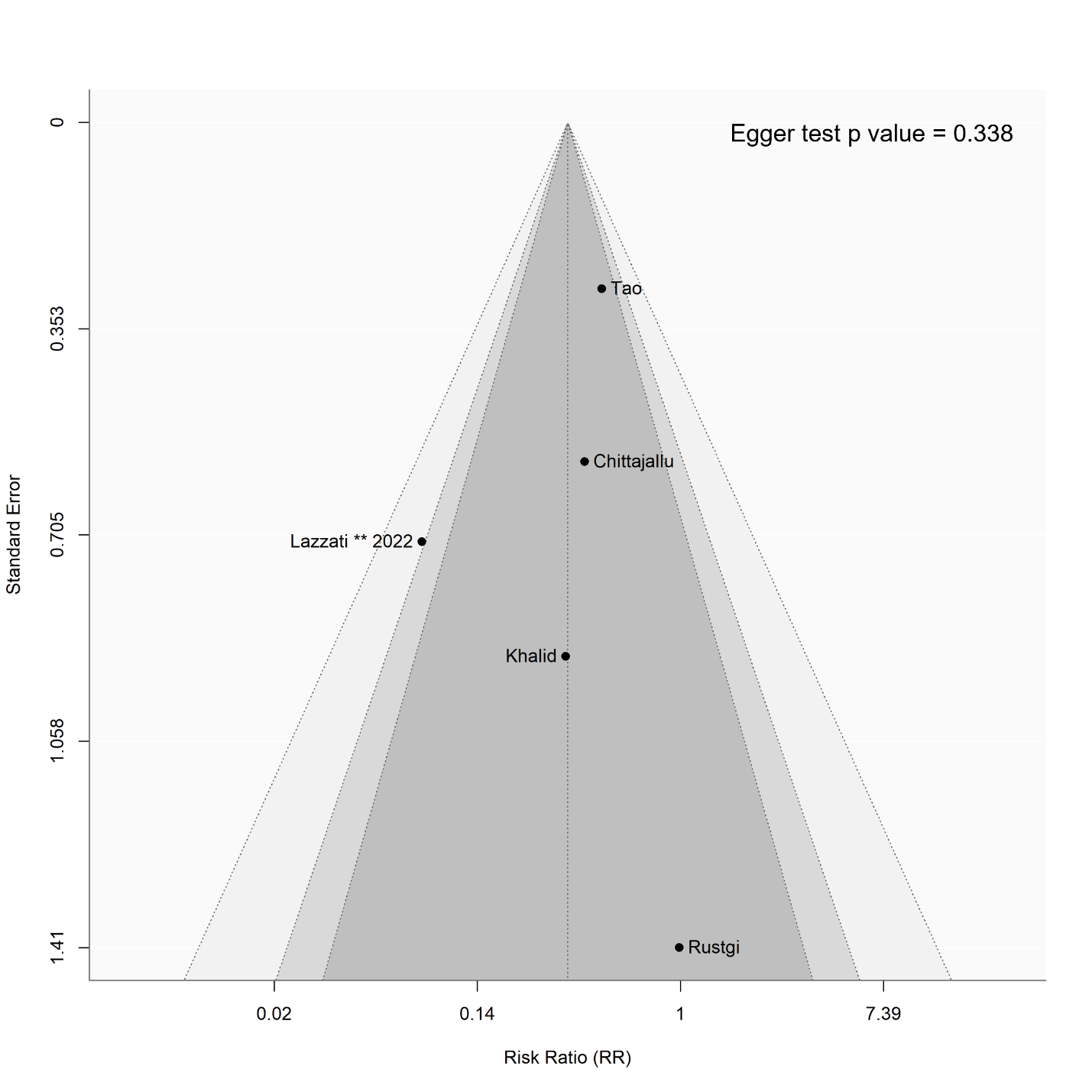
**

**Year, highlights which Lazzati paper is being presented (2022 or 2023)
